# Diversity of Phytoplasmas Infecting Plants and Insects in Iran Reveals Two Novel Ribosomal Subgroups

**DOI:** 10.3390/insects17020223

**Published:** 2026-02-21

**Authors:** Valeria Trivellone, Wardah Noor Syeda, Maryam Ghayeb Zamharir, Christopher H. Dietrich

**Affiliations:** 1 Illinois Natural History Survey, Prairie Research Institute, University of Illinois at Urbana-Champaign, Champaign, IL 61820, USAchdietri@illinois.edu (C.H.D.); 2 Plant Diseases Department, Iranian Research Institute of Plant Protection, Agricultural Research, Education and Extension Organization (AREEO), Tehran P.O. Box 19395-1454, Iran

**Keywords:** next-generation sequencing, planthoppers, heteroptera, multilocus tree, protein-coding genes, date palm, grapevine, soybean, barberry, olive

## Abstract

Phytoplasmas are obligate bacterial pathogens transmitted by phloem-feeding insects and responsible for severe diseases in numerous crops worldwide. In Iran, insect-associated phytoplasma transmission pathways remain poorly resolved, particularly at fine phylogenetic and vector-specific scales. Here, we investigated phytoplasma strains detected in four plant species, grapevine (*Vitis vinifera*), soybean (*Glycine max*), barberry (*Berberis vulgaris*), and the weed *Conyza canadensis*, and in three potential insect vectors (*Tropidocephala prasina*, *Eysarcoris ventralis*, and *Nysius graminicola*) collected from distinct agroecosystems across Iran. Phytoplasmas were characterized by using nearly full-length *16S rRNA* gene sequences and a multilocus dataset of protein-coding genes obtained through a targeted next-generation sequencing approach. Five phytoplasma strains belonging to ribosomal groups 16SrI, 16SrVI, 16SrIX, and 16SrXII were identified, including two novel ribosomal subgroups, 16SrI-AS and 16SrIX-K. Several previously unreported plant–phytoplasma and insect–phytoplasma associations were documented. Comparative phylogenetic analyses revealed that ribosomal and multilocus markers capture complementary evolutionary signals, with protein-coding genes providing additional resolution beyond 16S-based classification. These findings highlight the potential role of diverse hosts and polyphagous insects, not yet confirmed as vectors, in phytoplasma circulation and underscore how high-throughput next-generation sequencing and multilocus approaches advance our understanding of phytoplasma diversity and evolution.

## Simple Summary

Phytoplasmas are bacteria that cause serious diseases in many crops and are transmitted by sap-feeding insects. Iran hosts diverse agricultural systems and insect communities, yet insect–phytoplasma associations remain poorly investigated and incompletely documented. In this study, we investigated phytoplasma infections in economically and ecologically important plants, including grapevine, soybean, barberry, and weeds associated with olive orchards, and in potential insect vectors collected from date palm groves in Iran. Using targeted next-generation sequencing, we characterized phytoplasma strains based on the *16S rRNA* gene and multiple protein-coding genes. We detected five phytoplasma strains belonging to four ribosomal groups, including two previously undescribed subgroups. Several new plant–phytoplasma and insect–phytoplasma associations were identified, highlighting complex transmission pathways involving crops, weeds, and polyphagous insects. Our results show that combining ribosomal and multilocus analyses improves the resolution of phytoplasma diversity and provides insights into their ecology, evolution, and potential spread in agroecosystems.

## 1. Introduction

Phytoplasmas are a unique group of obligate pathogens inhabiting the phloem tissues of plants and the bodies of their insect hosts [1,2]. Unlike most bacteria, phytoplasmas lack cell walls and have undergone extensive reductive genome evolution, increasing their reliance on the host for multiplication and dispersal [3]. Phytoplasmas are responsible for a variety of plant diseases worldwide, affecting commodities, ornamentals and forestry, often causing symptoms such as yellowing, abnormal flower development, witches’ broom, stunting, and decline [4].

Phytoplasmas are currently classified primarily on the basis of *16S rRNA* gene sequence similarity using restriction fragment length polymorphism (RFLP) analysis, which assigns strains to ribosomal groups (16Sr groups) and subgroups [1]. Although this classification system has provided a stable framework for phytoplasma taxonomy and diagnostics, it relies on a highly conserved marker (approximately 1200 bp) and does not fully capture genome-wide evolutionary divergence, particularly among closely related strains or recently diverged lineages. Next-generation sequencing approaches have recently been proposed as an alternative to traditional methods for phytoplasma detection and identification, as they enable the recovery of longer *16S rRNA* gene sequences, increase detection sensitivity, and allow simultaneous characterization of additional genomic regions at relatively low cost, thereby providing greater discriminatory power for strain-level resolution [5].

Phytoplasmas are transmitted from plant to plant by phloem feeding insects in the order Hemiptera, mainly Auchenorrhyncha and Psyllidae [6], although a limited number of records also exist for heteropteran insects [7,8]. These insect vectors play an important role in the epidemiology of phytoplasma-associated diseases by maintaining high levels of inoculum across plant species and habitats. Because phytoplasmas are obligately dependent on their vectors for movement between host plants, host repertoire, and habitat colonization potential of each phytoplasma lineage are fundamentally constrained by the ecology and evolution of their vectors [9]. The ability of phytoplasmas to expand or contract their host ranges, spill over into new plant communities, or persist within stable transmission cycles depends on complex trophic interactions and the evolution of specific insect–phytoplasma–plant associations (e.g., see reviews and references therein [1,10]). Thus, investigations focusing on insect vectors are essential for understanding the mechanisms that drive phytoplasma spread, persistence, and diversification across natural and managed ecosystems.

Among phytoplasmas, certain groups are notoriously widespread and highly diversified. The phytoplasma group comprising ‘*Candidatus* (*Ca.*) Phytoplasma (P.) asteris’ [11] and ‘*Ca.* P. asteris’–related strains is one of the most cosmopolitan and diversified lineage, infecting hundreds of plant species and being transmitted by a broad array of leafhopper vectors. In many regions, its ecological success has been attributed to associations with highly mobile and polyphagous insects that facilitate long-distance dispersal and host shifts [12]. In contrast, closely related lineages such as the 16SrXII (Stolbur) phytoplasma group tend to exhibit more restricted geographic distributions and strong associations with specific families of leafhoppers and planthoppers. These patterns reflect complex interplays among vectors, host plants, and environmental conditions [13]. Other groups, including 16SrIX (Pigeon Pea Witches’ Broom) and 16SrVI (Clover Proliferation), further illustrate how vector ecology and habitat use shape phytoplasma diversity and evolutionary trajectories. For example, the genetic diversity observed among ‘*Ca*. P. phoenicium’-related strains has been suggested to arise from the adaptation of an ancestral strain to multiple hosts [14]. Altogether, these contrasting patterns underscore how vector ecology is a primary determinant of phytoplasma diversity, distribution, and emergence potential.

In Iran, phytoplasmas belonging to multiple ribosomal groups, including 16SrI, 16SrII, 16SrIII, 16SrVI, 16SrVII, 16SrIX, 16SrX, 16SrXI, 16SrXII, 16SrXIV, and 16SrXXIX, and 16SrXXX have been reported from a wide range of cultivated and wild plant hosts, such as grapevine, soybean, date palm, olive, barberry, and several weed species [15,16,17,18,19]. These phytoplasmas have been detected across diverse climatic regions, from humid to arid agroecosystems [16]. Despite the growing number of phytoplasma reports from plants, knowledge of insect–phytoplasma associations in Iran remains limited for several crops [7]. Only nine insect species have been experimentally confirmed as competent vectors (*Hishimonus phycitis*, *Orosius albicinctus*, *Exitianus capicola*, *Neoaliturus haematoceps*, *N. fenestratus*, *N. pulcher Austroagallia sinuata*, *Psammotettix striatus*, *Creontiades pallidus*) under controlled conditions [15,20,21,22,23,24,25,26], while most associations (~17) are inferred from field detection alone (e.g., [27]). This gap hampers a comprehensive understanding of phytoplasma epidemiology and the identification of key vectors responsible for its persistence and spread in Iranian agroecosystems. For example, grapevine, a crop of major economic and cultural importance in Iran, is affected by several ribosomal phytoplasma groups, including 16SrI, 16SrII, 16SrVII, 16SrIX, and 16SrXII [27,28]. Although multiple insect vectors have been implicated in this complex epidemiology, the specific transmission cycles have not yet been fully elucidated [29]. Olive is another iconic perennial crop that has been found infected with a ‘*Ca.* P. trifolii’-related strain [30]. Barberry, an economically important crop for Iran’s berry industry, was found to be infected with phytoplasma belonging to the 16SrII group [31]. Soybean was introduced into the Iranian agricultural landscape relatively recently, in 1939, and has been reported as infected with ‘*Ca.* P. trifolii’ (16SrVI-A) in 2018 [32]. *Creontiades pallidus* (Hemiptera: Miridae), a very common pest in soybean fields, has been confirmed as a competent vector under laboratory conditions [20].

In the present study, field surveys were conducted over a 10-year period to monitor phytoplasma diseases across six localities in Iran. Multiple agroecosystems including vineyards, date palm groves, soybean, barberry fields, and olive orchards, were sampled with a focus on abundant insect populations likely to play important epidemiological roles. In particular, one planthopper species, *Tropidocephala prasina* (family Delphacidae), and two heteropteran insects, *Eysarcoris ventralis* (family Pentatomidae) and *Nysius graminicola* (family Lygaeidae), were collected from date palm groves in Khuzestan Province with the aim of understanding the epidemiology of streak yellows disease. The planthopper *T. prasina* was found infected with ‘*Ca.* P. trifolii’ [33], highlighting its potential epidemiological relevance.

Here, we report new results obtained in 2025 using a next-generation sequencing approach based on phytoplasma-specific probe capture developed in the lead author’s laboratory at the University of Illinois [34]. The primary objective of this study was to characterize phytoplasma strains previously detected in these surveyed areas and to investigate the genetic diversity of phytoplasmas associated with abundant insect populations and key agroecosystems. By integrating targeted genomic enrichment with phylogenetic analyses, this work aims to refine phytoplasma strain characterization and contribute to a more robust understanding of phytoplasma–vector–plant relationships in Iran.

## 2. Materials and Methods

### 2.1. Study Area and Sample Collection

Sampling was conducted across multiple regions of Iran as part of independent epidemiological studies focusing on phytoplasma-associated diseases in agricultural and perennial systems, including soybean, date palm, grapevine, olive-associated weeds, and barberry. The study sites span northern (N), southern (S), northeastern (NE), and northwestern (NW) Iran (Table 1).

A total of 114 symptomatic grapevine (*Vitis vinifera*) samples were collected in July 2011 from Qazvin Province, one of the major viticultural regions of Iran. Sampling was conducted as part of a broader investigation into phytoplasma-associated grapevine diseases.

Insect sampling associated with phytoplasma-infected date palms was conducted in Abadan, Khuzestan Province, southern Iran. Sampling took place in groves with symptomatic date palms and targeted potential insect vectors. Insect sampling was designed to target abundant and ecologically prevalent sap-feeding taxa within the surveyed agroecosystems, selected for their potential epidemiological relevance in phytoplasma spread. Insects were collected using multiple methods, including sweep nets, manual shaking of insects from host plants, yellow sticky cards, and pan traps placed inside infected palm groves.

Two heteropteran species, *Eysarcoris ventralis* and *Nysius graminicola*, were collected from infected date palm groves in April 2017. In addition, the planthopper *Tropidocephala prasina* was collected in May 2017 using sweep nets and pan traps within the same agroecosystem. The insects were pooled in groups of 7–15 individuals, and a total of 9, 9 and 12 batches of E. *ventralis*, *N. graminicola*, and *T. prasina* respectively, were used for DNA extraction.

A total of 305 symptomatic soybean (*Glycine max*) samples and associated hemipteran insects were collected in northern Iran in 2020. Although phytoplasma infections were detected in both Mazandaran and Golestan provinces, analyses were restricted to samples from Mazandaran Province, and results presented here are limited to this province.

A total of 9 symptomatic samples of *Berberis vulgaris* were collected in June 2022 from cultivated fields in Birjand, South Khorasan Province, a major barberry-producing region in eastern Iran.

A total of 15 *Conyza canadensis* samples were collected in May 2019 from an olive orchard in Manjil, Gilan Province, where phytoplasma-infected olive trees were also present (independently identified as harboring mixed infections of ‘*Ca.* P. asteris’ and ‘*Ca*. P. fraxini’).

### 2.2. DNA Extraction and Phytoplasma Detection

Genomic DNA was extracted from plant leaf or whole insect body samples using a modified cetyltrimethylammonium bromide (CTAB) method based on Doyle and Doyle [35]. Briefly, 0.5–1.5 g of fresh leaf tissue or an insect pool was ground to a fine homogenate in 7.5 mL of preheated (60 °C) 2× CTAB extraction buffer (100 mM Tris-HCl, pH 8.0; 1.4 M NaCl; 20 mM EDTA; 2% CTAB; 0.2% β-mercaptoethanol). For recalcitrant tissues, sterile sand was added during grinding; alternatively, tissue was pulverized under liquid nitrogen prior to extraction. The homogenate was transferred to a 30 mL centrifuge tube, incubated at 60 °C for 30 min to allow complete cell lysis, and extracted once with chloroform–isoamyl alcohol (24:1, *v*/*v*) by gentle mixing. Phase separation was achieved by centrifugation at ~13,000× *g* for 10 min, and the aqueous phase was carefully transferred to a new tube using a wide-bore pipette. Nucleic acids were precipitated by adding two-thirds volume of cold isopropanol and mixing gently. The precipitated DNA was recovered either by spooling or centrifugation and washed for approximately 20 min in ethanol–ammonium acetate wash solution (76% ethanol, 10 mM ammonium acetate). Following washing, the DNA pellet was air-dried and resuspended in resuspension buffer (10 mM ammonium acetate, 0.25 mM EDTA) or TE buffer (10 mM Tris-HCl, pH 8.0; 1 mM EDTA). To remove RNA contamination, samples were treated with RNase A (final concentration 10 µg mL^−1^) and incubated at 37 °C for 30 min. DNA was subsequently re-precipitated by ethanol precipitation in the presence of ammonium acetate (final concentration 2.5 M), centrifuged at 10,000× *g* for 10 min, air-dried, and finally resuspended in TE buffer for downstream analyses.

To initially screen field-collected plant and insect samples for presence of phytoplasmas, PCR amplifications were performed using 20 ng of genomic DNA as template with the universal phytoplasma primer pair P1/P7 [36,37]. The resulting PCR products were diluted 1:30 and used as templates for nested PCR assays employing the primer pairs R16F2n/R2 [38]. A second nested PCR was performed on the nested PCR template using the primer pairs 16R758f/16R1232r (M1/M2) [39]. Phytoplasma-positive controls consisted of DNA extracted from phytoplasma reference strains maintained in almond, while reactions lacking DNA template were included as negative controls. PCR reactions were carried out in a total volume of 20 µL, containing 0.2 mM of each dNTP, 0.5 µL of each primer (20 pmol), 1 U of Taq DNA polymerase (Sinaclon, Karaj, Iran), and 1× Taq polymerase buffer. Thermal cycling conditions consisted of an initial denaturation at 94 °C for 1 min, followed by 35 cycles of denaturation at 94 °C for 1 min, annealing at 55 °C for 2 min, and extension at 72 °C for 2 min, with a final incubation step at 72 °C for 10 min. Following agarose gel electrophoresis, only samples yielding positive amplification results were selected for downstream sequencing analyses.

### 2.3. Anchored Hybrid Enrichment Sequencing and Bioinformatic Assembly

DNA templates of phytoplasma positive samples were analyzed using the Anchored Hybrid Enrichment (AHE) sequencing approach. This method uses custom-designed DNA hybridization probes to capture loci of interest from the phytoplasma genome, and sequence them using a next-generation platform. Preparation of AHE libraries and paired-end 120 bp sequencing were performed at LGC Genomics LLC (Middleton, WI, USA) using the probe kit which contains 57,914 probes mostly targeting phytoplasmas (178 loci from 129 genes). Raw sequencing reads were first processed using Clumpify (BBMap package, v39.06) to remove optical duplicates and reduce sequencing artifacts [40]. The quality of the cleaned reads was assessed using FastQC v0.12.1 [41]. High-quality reads were then assembled de novo using SPAdes 3.14.1 [42]. Assembled contigs were screened for target loci using TBLASTN v2.13.0, with protein sequences of the genes of interest used as queries and an E-value cutoff of 1 × 10^−5^. Candidate contigs were subjected to BLASTX searches against a curated reference database containing the original query sequences. Sequences showing reciprocal best BLAST hits were further validated by TBLASTN searches against the NCBI GenBank nucleotide database, and only sequences whose best hit corresponded to a phytoplasma gene were retained for downstream analyses. The *16S rRNA* gene was reconstructed from short-read assemblies and represents a consensus sequence. In phytoplasmas, multiple rrn operons may be present within a genome and can differ by a small number of nucleotides. As with direct Sanger sequencing of PCR amplicons and classical RFLP analyses, intragenomic variation among operons is therefore collapsed in the resulting sequence. Consequently, the reconstructed *16S rRNA* sequence should be interpreted as an operational taxonomic representation rather than an operon-specific haplotype. Sequencing quality control and coverage assessment were performed following the pipeline described in Trivellone et al. [34].

All the 16Sr RNA sequences were deposited in the National Center for Biotechnology Information (NCBI) database, with GenBank accession numbers PX767366-PX767372.

### 2.4. Blast Analysis and 16S Phytoplasma Classification

To identify and classify phytoplasma sequences, a dual-database approach was employed. All *16S rRNA* phytoplasma sequences were first queried against the NCBI nucleotide database using BLASTn v2.13.0 [43], and the closest matches were identified based on sequence similarity and alignment metrics. In addition, taxonomic assignment of 16Sr rRNA sequences was carried out using the *iPhyClassifier* https://plantpathology.ba.ars.usda.gov/cgi-bin/resource/iphyclassifier.cgi (accessed on 24 December 2025) platform [44], which applies internal classification modules specifically designed for phytoplasma 16Sr group and subgroup determination. For sequences representing putative new groups or subgroups, computer-simulated restriction fragment length polymorphism (RFLP) digestion of 16Sr rRNA sequences were conducted using the virtual gel module implemented in *iPhyClassifier*. Final taxonomic identifications were based on standard 16Sr *iPhyClassifier* criteria. Selected protein-coding gene sequences were characterized using BLASTn searches against the NCBI nucleotide database only.

### 2.5. Single-Gene (16S rRNA) and Multilocus Protein-Coding Phylogenetic Analyses

Phylogenetic inference was conducted separately for the *16S rRNA* gene and for a concatenated alignment of protein-coding genes. Multilocus phylogenetic analysis was performed using a concatenated dataset of 10 loci (*dnaK*, *ffh*, *ftsy*, *groES*, *groEL*, *grpE*, *map*, *rplO*, *rplP*, and *rplB*), with individual gene alignments concatenated using Concatenator v0.2.1 [45]. These loci encode essential cellular functions, are typically single-copy, and have been widely used in phytoplasma multilocus sequence analyses due to their balance between evolutionary conservation and phylogenetic informativeness. Only samples with complete recovery of all targeted loci were included in concatenated analyses to minimize missing data and ensure phylogenetic robustness.

A total of 32 and 16 phytoplasma reference strains, for *16S rRNA* and protein-coding genes respectively, were included in the analyses (Supplementary Tables S1 and S2). Sequence alignments were generated using MUSCLE 3.8.31 [46] as implemented in MEGA 11.0.13 [47].

Phylogenetic analyses were conducted using RAxML 2.0 (Randomized Axelerated Maximum Likelihood) program [48] under the GTR + Γ + I model of nucleotide substitution, with among-site rate heterogeneity modelled using a gamma distribution and the proportion of invariant sites estimated by maximum likelihood. A maximum-likelihood analysis with thorough bootstrapping was carried out using 1000 bootstrap replicates, with bootstrap support values mapped onto branch lengths. A single analysis run was conducted using a fixed random seed to ensure reproducibility, and *Acholeplasma laidlawii* was used as the outgroup to root the trees.

## 3. Results

### 3.1. Phytoplasma 16S rRNA-Based Identification

Overall, seven samples (four plants and three putative vectors) tested positive for phytoplasma during the initial PCR screening (Table 1). Anchored Hybrid Enrichment (AHE) assemblies yielded six nearly full-length *16S rRNA* (16Sr) phytoplasma sequences, ranging from 1246 to 1531 bp, whereas one heteropteran sample (*Nysius graminicola*, P002_WB05) yielded only a partial 16Sr fragment of 912 bp.

BLASTn and *iPhyClassifier* analyses provided congruent taxonomic assignments for most samples (Supplementary Table S3). BLASTn searches of partial to nearly full-length *16S rRNA* sequences (912–1531 bp) returned high-identity matches (99.47–100%), with coverage ranging from 95% to 100%, corresponding to ‘*Ca.* P. solani’, ‘*Ca.* P. phoenicium’, ‘*Ca.* P. trifolii’, and ‘*Ca.* P. asteris’. *iPhyClassifier* analyses confirmed these species-level assignments and further enabled 16Sr group and subgroup classification for six of the seven samples. Sequences from *V. vinifera* (P002_WA04) and *E. ventralis* (P002_WB04) were assigned to subgroups 16SrXII-A and 16SrVI-A, with 100% similarity with AF248959 (‘*Ca*. P. solani’) and AY390261 (‘*Ca.* P. trifolii’) respectively. Two samples from the plant hosts *G. max* (soybean) and *B. vulgaris* are both identical to the subgroup 16SrI-F reference strain (AY265211, ‘*Ca.* P. asteris’).

Virtual RFLP profiles were used as a comparative tool to assess consistency with previously defined phytoplasma subgroups, rather than as definitive operon-level representations. Two samples belonging to the 16SrI and IX groups exhibited collective RFLP profiles that differed from those of all previously established subgroups within their respective groups (Supplementary Figure S1). The sample from *C. canadensis* (P002_WC09) showed a distinct RFLP profile with restriction endonucleases *AluI*, *BfaI*, *EcoRI*, *HhaI*, *HinfI*, *HpaI*, and *Sau3AI.* The most similar strain was AY249247 (16SrI-AF), with a similarity coefficient of 0.88, and the phytoplasma detected was designated as a new subgroup, 16SrI-AS (Supplementary Figure S1A). Similarly, profiles from *AluI*, *BfaI*, *HhaI*, *HinfI*, *HpaI*, *Sau3AI*, *MseI* and *TaqI* digestions separated the strain from *T. prasina* sample (P002_WB03). The most similar strain was AF515637 (16SrIX-B), with a similarity coefficient of 0.93, and was designated as a new 16SrIX-K subgroup (Supplementary Figure S1B).

For one insect-derived sample (*N. graminicola*, P002_WB05), *iPhyClassifier* analysis was not possible due to insufficient sequence length; however, the short sequence showed high BLASTn identity to ‘*Ca.* P. trifolii’ and was identical to the sequence from *E. ventralis*.

### 3.2. Protein-Coding Gene Recovery and Multilocus Dataset

Recovery of phytoplasma protein-coding loci from AHE assemblies was evaluated across all seven PCR-positive samples and showed substantial variation among samples (Supplementary Table S4). Four samples (P002_WB03, P002_WB04, P002_WB08, P002_WC08) yielded no protein-coding gene sequences among the 10 genes selected. The insect-derived sample *N. graminicola* (P002_WB05; 16SrVI) yielded partial recovery, with three loci detected (*dnaK*, *ftsy*, and *groEL*). Complete recovery of all ten targeted protein-coding genes (*dnaK*, *ftsY*, *ffh*, *groES*, *groEL*, *grpE*, *map*, *rplB*, *rplO*, and *rplP*) was achieved for only two samples *V. vinifera* (16SrXII-A; P002_WA04, Markazi Province) and *C. canadensis* (16SrI-AS; P002_WC09, Gilan Province).

For the partially recovered sample *N. graminicola* (P002_WB05, 16SrVI), BLASTn searches of *dnaK*, *ftsY*, and *groEL* showed high similarity (98.15–100% identity) to phytoplasmas within the ‘*Ca.* P. asteris’ group, including Aster yellows witches’-broom phytoplasma (AYWB) and Potato purple top phytoplasma, although the 912 bp 16Sr sequence is identical to the ‘*Ca.* P. trifolii’ strain from *E. ventralis* (P002_WB04).

BLASTn characterization of protein-coding genes from *V. vinifera* revealed consistently high similarity (99.94–100% identity) to two ‘*Ca.* P. solani’ reference genomes (GenBank accessions CP103787–CP103788; FO393427–FO393428), indicating strong concordance with its 16SrXII-A classification (Supplementary Table S3). In contrast, protein-coding genes recovered from *C. canadensis* showed high but heterogeneous similarity (98.92–100% identity) to reference strains within the ‘*Ca.* P. asteris’ complex (16SrI), including Onion yellows phytoplasma OY-M (AP006628), ‘*Ca.* P. asteris’ strain M8 (CP128414), Mulberry dwarf phytoplasma (CP085837), and related strains such as Primula yellows (KJ494342.2) and Lettuce yellows phytoplasmas (AB242233). Virtual RFLP analysis of the *16S rRNA* gene using *iPhyClassifier* assigned the phytoplasma detected in *C. canadensis* to a novel subgroup, designated 16SrI-AS, despite high sequence similarity observed in individual protein-coding genes to previously described members of the 16SrI (‘*Ca.* P. asteris’) group based on BLASTn searches against the NCBI database.

### 3.3. Phylogenetic Tree Reconstruction Based on 16S rRNA Gene

**Figure 1:**
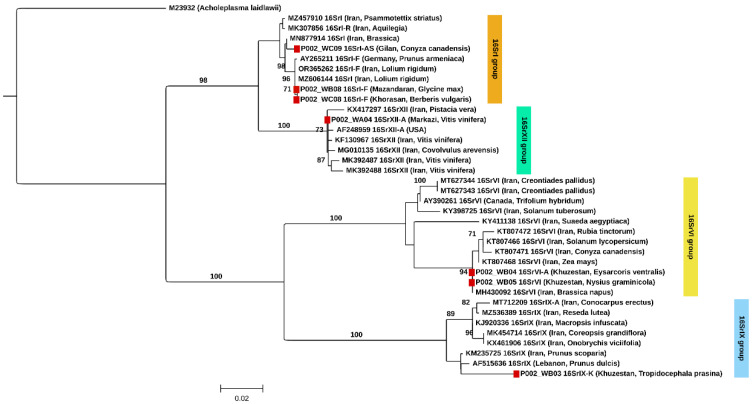
Phylogenetic tree inferred by Maximum Likelihood (ML) based on an alignment of 1848 nucleotide positions (including gaps) of the *16Sr rRNA* gene. Bootstrap values > 70% (1000 replicates) shown. Samples from this study are indicated with a red square. *Acholeplasma laidlawii* is the outgroup. Sample codes are provided in Supplementary Table S1.

Maximum-likelihood phylogenetic analysis of the *16S rRNA* gene was conducted using seven phytoplasma sequences generated in this study together with representative reference strains (Figure 1). The resulting topology was broadly consistent with BLASTn and *iPhyClassifier* assignments, with sequences clustering within their respective 16Sr groups and subgroups. Sequences obtained from *G. max* (P002_WB08, soybean), *B. vulgaris* (P002_WC08), and *C. canadensis* (P002_WC089) grouped within the 16SrI clade. The soybean and *B. vulgaris* samples, collected in Mazandaran and Khorasan Province, respectively, clustered together and formed a sister group of two reference strains belonging to 16SrI-F from Germany and Iran. The novel subgroup 16SrI-AS detected in *C. canadensis* collected in Gilan Province clustered as sister to a reference strain detected in *Brassica* sp. from Iran.

The phytoplasma detected in a sample of *V. vinifera* (P002_WA04) collected in Qazvin Province (Iran) grouped with reference strains of subgroup 16SrXII-A (‘*Ca*. P. solani’), including three strains previously reported from *V. vinifera* in Iran.

Phytoplasmas from two heteropteran insects, *E. ventralis* (P002_WB04) and *N. graminicola* (P002_WB05), both collected from the same locality in Khuzestan Province, clustered within subgroup 16SrVI-A (‘*Ca.* P. trifolii’) with strong bootstrap support (94%) and were identical to a reference strain detected on *Brassica napus* from Iran.

The novel subgroup 16SrIX-K detected in the planthopper *T. prasina* collected in Khuzestan Province grouped with two strains detected in *Prunus* spp. from Iran and Lebanon, although with a weak bootstrap support (<70%) within the 16SrIX clade. This subclade clustered with a larger group including reference strains of ‘*Ca.* P. phoenicium’.

### 3.4. Multilocus Phylogenetic Analysis Based on Protein-Coding Genes

**Figure 2:**
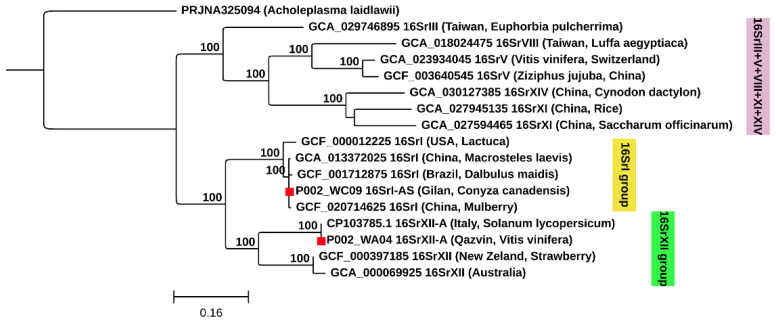
Phylogenetic tree inferred by Maximum Likelihood (ML) based on an alignment of 10,029 nucleotide positions (including gaps) of the 10 concatenated protein-coding genes (*dnaK*, *ffh*, *ftsy*, *groES*, *groEL*, *grpE*, *map*, *rplO*, *rplP*, and *rplB*). Bootstrap values > 70% (1000 replicates) shown. Samples from this study are indicated with a red square. *Acholeplasma laidlawii* is the outgroup. Sample codes are provided in Supplementary Table S2.

Multilocus phylogenetic inference based on concatenated protein-coding genes was performed for the subset of samples for which a complete gene set could be recovered (*V. vinifera* and *C. canadensis*) and included only reference strains with homologous loci were available in GenBank. Because the composition of reference strains necessarily differed from those of the samples used in the *16S rRNA*-based phylogenetic tree, direct topological comparisons between the two approaches should be made with caution. Nevertheless, the multilocus topology revealed coherent clustering patterns, placing the two samples within their respective phytoplasma lineages, i.e., the 16SrI group for *C. canadensis* and the 16SrXII group for *V. vinifera* (Figure 2).

The phytoplasma strain detected in *V. vinifera* grouped with the ‘*Ca*. P. solani’ reference strain (CP103785.1) and was recovered as sister to ‘*Ca.* P. australiense’-related strains, with strong bootstrap support (100%). The phytoplasma detected in *C. canadensis* clustered within the 16SrI group and was most closely related to two strains from China, detected in the leafhopper *Macrosteles laevis* and in *Paulownia* sp.

Overall, the multilocus analysis provided a well-resolved phylogenetic placement for both samples based on protein-coding genes.

## 4. Discussion

Iran is a major global producer of non-staple crops such as pistachio, dates, saffron, fruits, and raisins, making horticultural cultivation a cornerstone of its economy and rural livelihoods. Grapevine (*V. vinifera* L.) and olive (*Olea europaea*) are widely cultivated across multiple provinces, benefiting from the country’s ecological and climatic heterogeneity and long-standing traditional agricultural practices [49]. In particular, grapevine is grown extensively for fresh fruit and raisin production, contributing substantially to local economies in northern and western regions. Olive cultivation has expanded markedly in recent decades, with more than 100,000 hectares of olive groves supporting both table olive and olive oil industries and generating millions of workdays annually, underscoring its socio-economic importance beyond traditional Mediterranean regions [50]. *Berberis vulgaris*, locally known as zereshk, represents another culturally and economically significant crop, with Iran ranking among the world’s leading producers of dried barberry fruits used extensively in Persian cuisine and export markets. Soybean (*G. max*) is also gaining importance as part of national efforts to diversify agricultural production and meet increasing domestic and industrial demand. Several phytoplasma-associated diseases have been reported from these crops and their associated insect communities in Iran [29,30,31,32]. In this context, understanding the diversity, distribution, and evolutionary relationships of phytoplasmas affecting these agroecosystems is essential for improving plant health management strategies and safeguarding agricultural productivity.

In this study, we report five distinct phytoplasma lineages, including three previously described groups and two novel ribosomal subgroups, detected across three cultivated crops (grapevine, soybean, and barberry), one weed species associated with olive agroecosystems, and three putative insect vectors. We confirmed one previously known host association (grapevine-‘*Ca.* P. solani’) and identified six novel phytoplasma-host associations involving three plant species (soybean-16SrI-F; barberry-16SrI-F; *C. canadensis*-16SrI-AS) and three insect species (*T. prasina*-16SrIX-K; *E. ventralis*-‘*Ca.* P. trifolii’; *N. graminicola*-16SrVI).

Grapevine is known to host six different phytoplasma groups in Iran (16SrI, 16SrII, 16SrVI, 16SrVII, 16SrIX, and 16SrXII) [28,51,52]. In this study, we detected ‘*Ca.* P. solani’ in a grapevine sample collected in Markazi Province. Nearly full-length *16S rRNA* sequences of ‘*Ca.* P. solani’ have previously been reported from grapevine in Fars (southern Iran) and Lorestan (western Iran) [53] and differed from our sequence by only three single-nucleotide polymorphisms (SNPs) across 86% sequence coverage. In contrast, shorter sequences (879 bp; 57% coverage) reported by Ghayeb Zamharir et al. [28] showed greater divergence, including 17 SNPs. The close similarity between the nearly full-length *16S rRNA* sequence obtained in this study and previously reported Iranian grapevine isolates of ‘*Ca.* P. solani’ suggests the presence of a relatively conserved lineage circulating across geographically distant viticultural regions in Iran [54,55]. Moreover, the recurrence of closely related ‘*Ca.* P. solani’ strains in grapevine across multiple provinces supports the hypothesis of stable host association and possible long-distance dissemination through infected planting material or shared insect vectors.

Soybean (*G. max*), barberry (*B. vulgaris*), and a weed (*C. canadensis*) associated with olive orchards were found infected with phytoplasmas belonging to the 16SrI group. In Iran, soybean had previously been reported to be associated with ‘*Ca.* P. trifolii’ (16SrVI-A) [20,32], whereas phytoplasmas of subgroup 16SrI-F have been recorded from a range of other hosts, including *Tamarix aphylla*, *Gladiolus* spp., *Catharanthus roseus*, and *Lolium rigidum* [17,56,57,58]. The detection of 16SrI-F in soybean and barberry from this study therefore represents an expansion of the known host range of this subgroup within Iranian agroecosystems. As reported by Esmaeilzadeh-Hosseini et al. [17], the wide host range of 16SrI-F phytoplasmas suggests a high potential for epidemiological connectivity among herbaceous plants, perennial crops, and weeds. The presence of this subgroup in taxonomically and ecologically diverse hosts supports the hypothesis that transmission may be mediated by polyphagous insect vectors capable of exploiting multiple plant species. Moreover, the occurrence of 16SrI-F in crops and non-crop hosts raises concern about its possible dissemination through infected propagation material, particularly in nursery and orchard settings, where asymptomatic infections may facilitate unnoticed spread. Together, these findings underscore the need for integrated surveillance approaches that include weeds, insect vectors, and planting material to better assess phytoplasma epidemiology and manage disease risks in Iranian agricultural systems.

In Iran, *C. canadensis* had previously been reported to be associated with ‘*Ca.* P. trifolii’ (16SrVI-A) in West Azerbaijan Province, where it was collected in urban areas [59]. Here, we report a new association of *C. canadensis* with a novel phytoplasma subgroup, designated 16SrI-AS. In the present study, *C. canadensis* occurred as a weed growing within an olive orchard. This finding is epidemiologically relevant, as *C. canadensis* may act as a potential reservoir of inoculum for the infection of olive trees.

In 2013, Ghayeb Zamharir and Arbabtafti [33] reported palm leaf streak disease associated with phytoplasma infection in date palms from several provinces in Iran, including Khuzestan Province. In the same province, they also collected the planthopper *T. prasina* and found it to be infected with ‘*Ca.* P. trifolii’ (16SrVI-A). In the present study, the same insect species, collected in Khuzestan Province in 2017, was instead found to be infected with a novel phytoplasma subgroup, designated 16SrIX-K. In Iran, several phytoplasma groups have been reported in association with date palm, including 16SrVI-A and 16SrVII-A, highlighting the diversity of phytoplasmas circulating within date palm agroecosystems [33,60,61].

In this study, the heteropteran insects *E. ventralis* and *N. graminicola* both collected in Khuzestan Province, were found to harbor ‘*Ca.* P. trifolii’ (16SrVI-A). Phytoplasmas belonging to the 16SrVI group have previously been reported in Iran from both plant and insect hosts. Notably, Zamharir and Shameli [7] detected ‘*Ca.* P. trifolii’ in the mirid bug *Creontiades pallidus* collected on soybean, supporting the circulation of this phytoplasma lineage within agroecosystems involving leguminous crops and associated insects. In addition, *E. ventralis* and *N. graminicola* were previously recorded by Zamharir and Arbabtafti [33] in date palm groves in Khuzestan Province, although phytoplasma identity was not assessed in those earlier surveys. The detection of ‘*Ca.* P. trifolii’ in these heteropteran species suggests that they may acquire phytoplasmas while feeding on infected plants within mixed-crop or semi-natural habitats. Given the polyphagous feeding behavior of many heteropterans and their frequent occurrence in agricultural landscapes, these insects could contribute to the local maintenance and spread of 16SrVI phytoplasmas. Detection of phytoplasmas in insects is challenging because, unlike plants, insects do not exhibit symptoms, and detection represents only a temporal snapshot without indication of the infection stage. Consequently, the presence of phytoplasma DNA alone cannot distinguish competent vectors from incidental or transient hosts, underscoring the need for complementary approaches, including transmission assays, tissue localization, and analyses of temporal infection dynamics. For these reasons, the potential role of these insects as competent vectors remains to be experimentally demonstrated.

Nearly full-length *16S rRNA* gene sequences were recovered for all but one sample, whereas recovery of protein-coding loci varied substantially among samples, with complete retrieval of all ten targeted genes achieved for only two strains. This uneven recovery likely reflects a combination of factors, including locus absence or divergence among phytoplasma strains, variability in capture efficiency inherent to the AHE approach, as well as differences in DNA quality. In addition, the relatively sparse availability of homologous protein-coding genes in public databases constrained the selection of reference strains for multilocus analyses, resulting in partial differences between the reference sets used for the *16S rRNA* and concatenated protein-coding phylogenies.

These constraints complicate direct comparisons between single-locus and multilocus trees and may influence relative branch lengths and perceived divergence. Nonetheless, the multilocus phylogeny revealed consistent and biologically meaningful patterns that are unlikely to be explained solely by reference sampling effects, supporting the presence of genuine genome-wide differentiation among the analyzed phytoplasma lineages.

Within the 16SrI lineage, the phytoplasma detected in *C. canadensis* exhibited relatively long branch lengths and increased divergence from reference strains in the *16S rRNA* phylogeny, consistent with its designation as a novel ribosomal subgroup (16SrI-AS) based on *iPhyClassifier* analysis. In contrast, the multilocus phylogeny based on concatenated protein-coding genes placed the same strain in closer proximity to related taxa, suggesting that divergence at the *16S rRNA* locus may accentuate ribosomal differentiation without necessarily reflecting broad genomic divergence. Conversely, within the 16SrXII lineage, the *16S rRNA* gene showed high similarity between the *Vitis vinifera* strain and Iranian reference strains belonging to the same subgroup (16SrXII-A). In the multilocus analysis, however, divergence was more pronounced because the closest available reference genomes belonged to a different subgroup (16SrXII-B).

Taken together, these results emphasize that *16S rRNA*-based classification and multilocus phylogenetic reconstruction capture complementary aspects of phytoplasma diversity. Ribosomal subgroup assignments provide a standardized and essential framework for phytoplasma taxonomy, while multilocus analyses offer additional resolution for exploring genome-wide relationships, host-associated divergence, and evolutionary processes. Accordingly, the designation of the novel subgroup 16SrI-AS should be interpreted as a formal ribosomal classification within the ‘*Ca.* P. asteris’ complex, without implying extensive genomic divergence beyond that captured by the *16S rRNA* gene alone.

## 5. Conclusions

This study expands the known diversity of phytoplasmas infecting plants and insects in Iran by identifying two novel ribosomal subgroups and documenting new host–pathogen associations. While *16S rRNA*-based classification remains essential for subgroup designation, multilocus analyses provided complementary resolution and clarified phylogenetic relationships among closely related lineages, although further pointing out the need to expand genome resources to improve reference representation in future studies. The detection of ‘*Ca.* P. trifolii’ (16SrVI-A) in multiple heteropteran species, including previously unreported associations, highlights heteropteran insects as candidates for further investigation, while acknowledging that detection alone does not demonstrate epidemiological relevance or vector competence. Together, these findings highlight the importance of integrating ribosomal and protein-coding markers with insect host data to improve phytoplasma taxonomy, epidemiological inference, and our understanding of pathogen circulation across agro-ecosystems.

## Figures and Tables

**Table 1:** List of samples collected in five locations in Iran that tested positive for the presence of phytoplasmas and were successfully sequenced using Anchored Hybrid Enrichment (AHE) approach.

Sample Code	Host Species	Date Collection	Coordinates	Locality ^1^
P002_WA04	*Vitis vinifera* (grapevine)	31 July 2011	35°21′29.9″ N 50°25′50.9″ E	Takestan, Qazvin (N)
P002_WB03	*Tropidocephala prasina*	16 May 2017	30°50′34.1″ N 48°28′51.6″ E	Abadan, Khuzestan (S)
P002_WB04	*Eysarcoris ventralis*	15 April 2017	30°44′40.9″ N 48°25′52.0″ E	Abadan, Khuzestan (S)
P002_WB05	*Nysius graminicola*	9 April 2017	30°44′40.9″ N 48°25′52.0″ E	Abadan, Khuzestan (S)
P002_WB08	*Glycine max* (soybean)	26 September 2020	36°22′28.9″ N 53°25′52.0″ E	Mazandaran (N)
P002_WC08	*Berberis vulgaris* (barberry)	30 June 2022	32°52′23.9″ N 59°12′58.7″ E	Birjand, South Khorasan (NW)
P002_WC09	*Conyza canadensis*	26 May 2019	36°45′32.8″ N 49°23′16.8″ E	Manjil, Gilan (NE)

^1^ Locality, Province (N, northern; S, southern; NE, northeastern; NW, northwestern).

## Data Availability

All the 16Sr RNA sequences were deposited in the National Center for Biotechnology Information (NCBI) database, with GenBank accession numbers PX767366-PX767372. The raw reads from AHE are available from the first author upon request.

## Supplementary Materials

The following supporting information can be downloaded at: https://www.mdpi.com/article/10.3390/insects17020223/s1, Figure S1: Distinct virtual RFLP patterns from in silico digestion of *16Sr* gene F2nR2 fragments of the two sequences belonging to 16SrIX and 16SrI phytoplasma groups detected in this study. (A) Sample P002_WB03, 16SrIX-K detected in *Tropidocephala prasina*; (B) Sample P002_WC09, 16SrI-AS detected in *Conyza canadensis*. Recognition sites for the following 17 restriction enzymes were used in the simulated digestions: *AluI*, *BamHI*, *BfaI*, *BstUI* (ThaI), *DraI*, *EcoRI*, *HaeIII*, *HhaI*, *HinfI*, *HpaI*, *HpaII*, *KpnI*, *Sau3AI* (cMboI), *MseI*, *RsaI*, *SspI*, and *TaqI*. MW, ϕ X174 DNA-HaeIII digestion as a marker; Table S1: List of Reference strains used to build the 16S rRNA phylogenetic tree; Table S2: List of Reference strains used to build the multilocus phylogenetic tree; Table S3: Dual-Database sequence analysis and phytoplasma classification based on 16S rRNA gene and 10 protein-coding genes; Table S4: Recovery of protein-coding phytoplasma genes across AHE assemblies. Presence (yes) or absence (–) of each locus is shown for the seven phytoplasma-positive samples. Only samples with complete recovery of all targeted loci were included in the multilocus phylogenetic analysis. References [62,63,64,65,66,67,68] are cited in the supplementary materials.
