# Supplementary material for: Diversity of Phytoplasmas Infecting Plants and Insects in Iran Reveals Two Novel Ribosomal Subgroups
Source: Insects. 2026 Feb 21;17(2):223. doi: 10.3390/insects17020223 (PMC12941973; doi:10.3390/insects17020223)
Supplement: Supplementary file 1 [file insects-17-00223-s001.zip › Supplementary Figure S1.pdf]

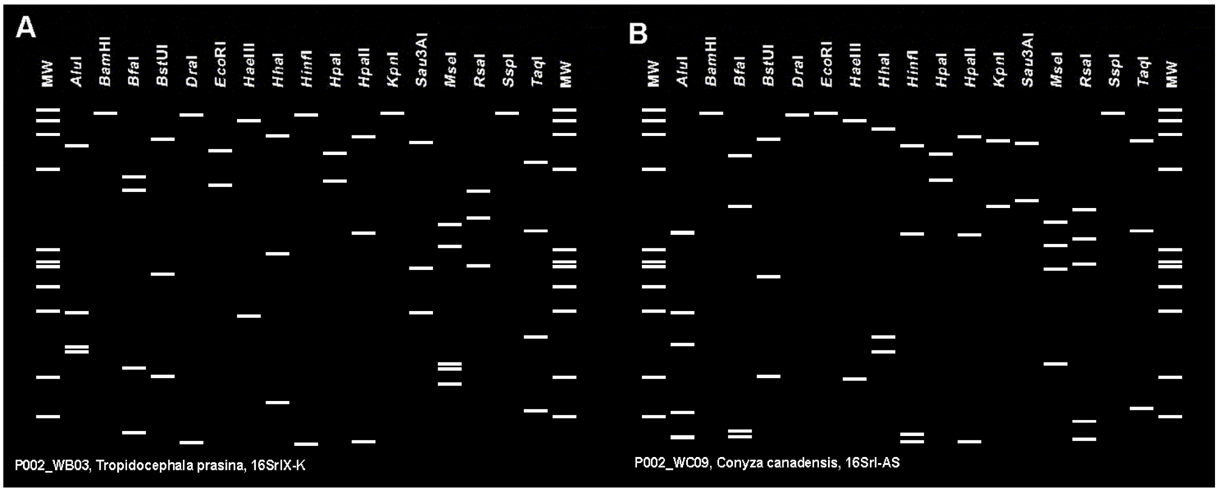

Figure S1. Distinct virtual RFLP patterns from in silico digestion of *16Sr* gene F2nR2 fragments of the two sequences belonging to 16SrIX and 16SrI phytoplasma groups detected in this study. (A) Sample P002_WB03, 16SrIX-K detected in *Tropidocephala prasina*; (B) Sample P002_WC09, 16SrI-AS detected in *Conyza canadensis*. Recognition sites for the following 17 restriction enzymes were used in the simulated digestions: *AluI, BamHI, BfaI, BstUI (ThaI), DraI, EcoRI, HaeIII, HhaI, HinfI, HpaI, HpaII, KpnI, Sau3AI* (cMboI), *MseI, RsaI, SspI, and TaqI*. MW, ϕ X174 DNA-HaeIII digestion as a marker.
